# Isolation of biologically active peptides from the venom of Japanese carpenter bee, *Xylocopa appendiculata*

**DOI:** 10.1186/s40409-017-0119-6

**Published:** 2017-05-23

**Authors:** Hiroko Kawakami, Shin G. Goto, Kazuya Murata, Hideaki Matsuda, Yasushi Shigeri, Tomohiro Imura, Hidetoshi Inagaki, Tetsuro Shinada

**Affiliations:** 1 Graduate School of Material Science, Osaka City University, 3-3-138 Sugimoto, Sumiyoshi, Osaka 558-8585 Japan; 2 Graduate School of Science, Department of Biology & Geosciences, Osaka City University, 3-3-138 Sugimoto, Sumiyoshi, Osaka 558-8585 Japan; 3 Faculty of Pharmacy, Kindai University, 3-4-1 Kowakae, Higashiosaka, Osaka 577-8502 Japan; 4 Health Research Institute, National Institute of Advanced Industrial Science and Technology (AIST), Osaka, Japan; 5 Research Institute for Chemical Process Technology, National Institute of Advanced Industrial Science and Technology (AIST), Ibaraki, Japan; 6 Biomedical Research Institute, National Institute of Advanced Industrial Science and Technology (AIST), Ibaraki, Japan

**Keywords:** *Xylocopa appendiculata*, Carpenter bee, Venom peptides, Solitary bee, Mass spectrometry analysis

## Abstract

**Background:**

Mass spectrometry-guided venom peptide profiling is a powerful tool to explore novel substances from venomous animals in a highly sensitive manner. In this study, this peptide profiling approach is successfully applied to explore the venom peptides of a Japanese solitary carpenter bee, *Xylocopa appendiculata* (Hymenoptera: Apoidea: Apidae: Anthophila: Xylocopinae: Xylocopini). Although interesting biological effects of the crude venom of carpenter bees have been reported, the structure and biological function of the venom peptides have not been elucidated yet.

**Methods:**

The venom peptide profiling of the crude venom of *X. appendiculata* was performed by matrix-assisted laser desorption/ionization-time of flight mass spectroscopy. The venom was purified by a reverse-phase HPLC. The purified peptides were subjected to the Edman degradation, MS/MS analysis, and/or molecular cloning methods for peptide sequencing. Biological and functional characterization was performed by circular dichroism analysis, liposome leakage assay, and antimicrobial, histamine releasing and hemolytic activity tests.

**Results:**

Three novel peptides with *m*/*z* 16508, 1939.3, and 1900.3 were isolated from the venom of *X. appendiculata*. The peptide with *m*/*z* 16508 was characterized as a secretory phospholipase A_2_ (PLA_2_) homolog in which the characteristic cysteine residues as well as the active site residues found in bee PLA_2_s are highly conserved. Two novel peptides with *m/z* 1939.3 and *m/z* 1900.3 were named as Xac-1 and Xac-2, respectively. These peptides are found to be amphiphilic and displayed antimicrobial and hemolytic activities. The potency was almost the same as that of mastoparan isolated from the wasp venom.

**Conclusion:**

We found three novel biologically active peptides in the venom of *X. appendiculata* and analyzed their molecular functions, and compared their sequential homology to discuss their molecular diversity. Highly sensitive mass analysis plays an important role in this study.

**Electronic supplementary material:**

The online version of this article (doi:10.1186/s40409-017-0119-6) contains supplementary material, which is available to authorized users.

## Background

The venom of bees (Hymenoptera: Apoidea: Anthophila) such as honeybees (Hymenoptera: Apoidea: Apidae: Anthophila: Apinae: Apini) and bumblebees (Apoidea: Apidae: Anthophila: Apinae: Bombini) has attracted significant attention as rich sources of biologically active peptides [1, 2]. Extensive isolation and biological studies on bee venom have disclosed that it is composed of various biologically active molecules: biogenic amines, peptides and enzymes. Apamine, MCD-peptide, melittin [3], bombolitins [4], phospholipase A_2_ (PLA_2_), and hyaluronidase [5] are representative peptide components isolated from the venom of the honeybee *Apis mellifera* [6] and bumblebees. These peptides reveal a broad range of biological activities such as mast cell degranulating, antimicrobial, histamine releasing, and/or inflammatory activities, and were speculated as toxic principles to cause severe pain [1–6]. In contrast, the venom has been utilized in folk medicine to cure various diseases for long time. Recently, its potential has been revisited [7, 8].

**Fig. 1:**
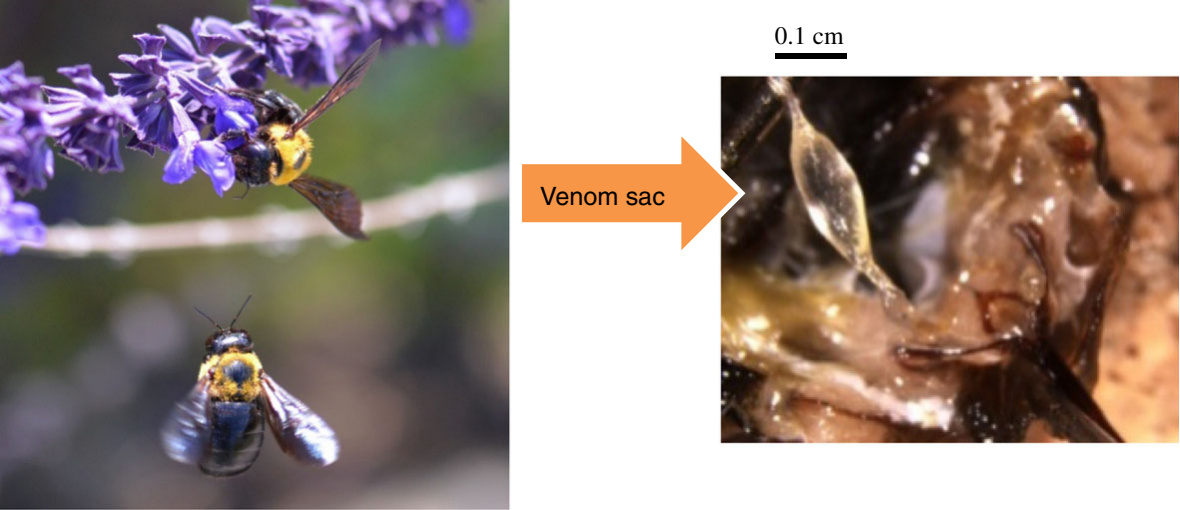
*Xylocopa appendiculate* and its venom sac. The adults are approximately 20-25 mm in length, The image of *Xylocopa appendiculate* is kindly provided by Professor Hideharu Numata (Graduate School of Science, Kyoto University)

Mass spectrometry-guided venom peptide profiling has become an indispensable tool for rapid, accurate, and highly sensitive screening of the novel venom substances [9–12]. It contribute to accelerate the elucidation of the venom substances in the molecular structure level. Recently, we have successfully applied mass spectrometry-guided venom peptide profiling to explore novel venom substances of social and solitary wasps [13, 14]. In conjunction with our continuous research program on the isolation and biological study of the Hymenoptera venom substances, we were interested in the venom of the Japanese carpenter solitary bee *Xylocopa appendiculata* (Hymenoptera: Apoidea: Apidae: Anthophila: Xylocopinae: Xylocopini). We considered that the target venom is a challenging sample because of the following reasons:

- the crude venom of carpenter bees showed significant biological effects such as lethal activities in a small bird and mice [15];
- the sting of *Xylocopa virginica* and *Xylocopa vioracea* seems to be as painful in humans as are honeybee stings [15];
- although the significant biological effects of the crude venom has been suggested, the biologically active peptides of the carpenter bee venom including that of *X. appendiculata* has not been isolated yet;
- it is difficult to collect *X. appendiculata* because of their solitary lives; and
- only a small amount of the venom substances is available due to the fact that the venom sac of *X. appendiculata* is smaller than those of honeybees and vespid wasps (Fig. 1).

## Methods

### Sample preparation

Fifteen female bees of *X. appendiculata* were collected in Osaka and Sakai, Japan. The venom sacs were dissected and homogenized with water (50 μL). The venom extract was applied to MALDI-TOF MS and HPLC analyses.

### MALDI-TOF MS and MS/MS analysis

Matrix-assisted laser desorption/ionization-time of flight mass spectroscopy (MALDI-TOF MS) and tandem mass spectrometry (MS/MS) analysis was performed by UltraFlex speed (Bruker Daltonics, Germany). MS and MS/MS analyses (Additional files 1 and 2) were performed in the linear positive ion mode and reflector positive mode, respectively. α-Cyano-4-hydroxycinnamic acid (CHCA), trifluoroacetic acid (TFA) and all other reagents were purchased from Wako Pure Chemical Industries, Ltd. (Osaka, Japan) or Nacalai Tesque (Kyoto, Japan). The matrix solution was prepared as follows. CHCA was dissolved in 3:7 acetonitrile/H_2_O (0.1% TFA) to obtain saturated matrix solution. The crude venom or peptide sample were mixed with the matrix solution on a plate, dried for 5 min at ambient temperature, and provided for the mass analysis. Molecular mass and peptide sequencing were analyzed using the FlexAnalysis 3.4 software and BioTools 3.2 (Bruker Daltonics, Germany). The monoisotopic molecular mass was estimated in a range of *m*/*z* 1000 ~ 5000 for short peptides or *m*/*z* 5000 ~ 20,000 for PLA_2_.

### High performance liquid chromatography (HPLC) analysis

HPLC analysis and purification of the crude venom was performed by Shimadzu’s Prominence system (Japan). Chromatographic conditions for analysis were as follows:

- Column: COSMOSIL 5C18-AR-300, 4.6 mm × 150 mm (Nacalai Tesque).
- Eluent: (I) CH_3_CN containing 0.1% TFA and (II) H_2_O containing 0.1% TFA.
- Elution: linear gradient from (I):(II) = 0.1:99.9 to 60:40.
- Flow rate: 1.0 mL/min for 45 min.
- Detection: UV 210 nm.

Whereas the conditions for purification were:

- Column: COSMOSIL Protein-R, 4.6 mm × 250 mm (Nacalai Tesque).
- Eluent: (I) CH_3_CN containing 0.1% TFA and (II) H_2_O containing 0.1% TFA.
- Elution: linear gradient from (I):(II) = 0.1:99.9 to 60:40.
- Flow rate: 1.0 mL/min for 45 min.
- Detection: UV 210 nm.

### Peptide sequence analysis and synthesis

The purified venom peptides were sequenced by automated Edman degradation using ABI model 477A (Applied Biosystems, USA). Peptides were synthesized by Fmoc chemistry using a Shimadzu PSSM-8 automated peptide synthesizer (Shimadzu, Japan), and purified by reverse-phase HPLC. The identity and purity of the peptides were confirmed by MALDI-TOF MS. The synthetic Xac-1 and Xac-2 were employed for circular dichroism (CD) analysis, liposome leakage assay, antimicrobial and hemolytic activity tests.

### Database search

Peptide database search of the venom peptides was implemented by using NCBI database (http://www.ncbi.nlm.nih.gov/) and Hymenoptera Genome Database (http://hymenopteragenome.org/).

### Circular dichroism (CD) analysis

CD analysis was performed by a spectropolarimeter (J-720 W; JASCO) at room temperature. Spectra were obtained at wavelength 190-260 nm. Four scans were accumulated for each sample at a scan rate of 20 nm/min. The synthetic peptides were measured at concentration of 0.2 mM in H_2_O and 50% (v/v) trifluoroethanol (TFE)/H_2_O.

### Liposome leakage experiments

Liposomes were prepared from lecithin from egg yolk (phosphatidylcholine approx. 70%; Nacalai Tesque). The lecithin (28 mg) was dissolved in chloroform (5 mL). The solution was concentrated in vacuo and maintained under the reduced pressure for 10 h to remove the solvent. The dried lecithin was hydrated in 4 mL of 70 mM calcein (Sigma-Aldrich) in aqueous NaOH (pH 7.5). After sonication for 10 min, the vesicles were passed through a column of Sephadex^TM^ G-50 (GE Healthcare) in H_2_O to remove free calcein. The first 5 mL of eluent was collected as calcein-encapsulated vesicles. Water (0.8 mL) was added to the liposome suspension (0.2 mL) in a cuvette. After 10 min, 0.5-20 μL of 10 mM solution of mastoparan (Peptide Institute, Inc., Japan) or Xac-1 was added to the cuvette. Fluorescence intensity of calcein was measured by Hitachi P-4500 fluorometer (excitation wavelength of 460 nm and emission wavelength of 530 nm). A 1% (v/v) solution of Triton X-100 was used as a positive control to obtain maximum fluorescent value at 100% leakage of calcein.

### Molecular cloning

RNA was extracted from the venom gland and the venom sac by Trizol reagent (Life Technologies, USA). cDNA was synthesized with oligo(dT)_12-18_ primer and M-MLV reverse transcriptase (Life Technologies). Degenerate primers were designed on the basis of the nucleotide sequences of PLA_2_ genes of several Hymenopteran species. PCR was performed with the cDNA by using Xc2 (5′-AAY GGI AAY GTN GCN GAR GG-3′) and Xc4 (5′-AVR TCR AAC CAY TGR TA-3′) primers, and subsequently the nested PCR was performed with the first PCR product as a template by using Xc2 and Xc3 (5′-GCN GAR GGI CCN GAR GAY-3′) primers.

PCR products were cloned into plasmids using pGEM-T Easy Vector System (Promega, USA). Plasmids were purified with Wizard Plus SV Minipreps DNA Purification System (Promega) and sequenced on an ABI PRISM 310 Genetic Analyzer (Life Technologies) or 3130 Genetic Analyzer (Life Technologies) with BigDye Terminator v3.1 Cycle Sequence kit (Life Technologies). To obtain complete sequences of PLA_2_ cDNA, 3′- and 5′-RACEs (rapid amplification of cDNA ends) were performed using a SMART RACE cDNA Amplification kit (Clontech, USA) according to the supplier’s instructions. F3 (5′-CGG CGC CGT AAG GTT CAC GTA CTT C) and R1 (5′-GCT GAA GGA GAC CGA CGC CTG TTG T-3′) primers were used for 3′- and 5′ RACEs, respectively. The obtained PCR products were also cloned into a vector, and sequenced as described above.

### Antimicrobial activity

According to the procedure [16, 17], antimicrobial activities of Xac-1 and Xac-2 were evaluated using *Escherichia coli* (NBRC14237) and *Micrococcus luteus* (NBRC 12708) as a gram-negative bacterium, *Stapylococcus aureus* (NBRC12708) as a gram-positive bacterium, and the yeast *Saccharomyces cerevisiae* (NBCR 10217). To compare the potency, MIC values of mastoparan were evaluated. Bacteria were grown in 2 mL Trypticase soy broth, and yeasts in Sabouraud dextrose broth for 16 h with shaking at 200 rpm as a pre-culture. Subsequently, 0.1 mL pre-culture medium was inoculated into 2 mL of fresh medium. It was cultivated for 2-3 h until A_600_ = 0.5. The cultivated medium was diluted with PBS solution. The diluted microbial broth (100 μL) was mixed with peptide solutions (11 μL) in 96-well plates and incubated for 3 h. After 3 h incubation, two times concentrated medium were added and 96-well plates were reincubated for 16 h. Microbial growth was measured by Spectra MAX 190 microplate reader at A_600_.

### Hemolytic activity

According to the procedure described by Shigeri et al. [12], hemolytic activities of Xac-1 and Xac-2 were tested. Heparinized rat whole blood from Wistar rats (male, 6 weeks old) was washed twice in NaCl/Pi (100 mM NaCl, 7.5 mM Na_2_HPO_4_ and 2.5 mM NaH_2_PO_4_) by centrifugation at 900 g and suspended in NaCl/Pi to a concentration of 0.5% (v/v). NaCl/Pi and NaCl/Pi containing 0.2% Triton X-100 were used as controls for 0 and 100% hemolysis, respectively. Xac-1 and Xac-2, as well as mastoparan and melittin were employed as comparable standards.

### Histamine releasing activity

The histamine-releasing activities of Xac-1 and Xac-2, mastoparan, and melittin were determined with rat peritoneal mast cells, as previously described [17]. The histamine-releasing activity was defined as the ration of the extracellular to the total amount of histamine. Spontaneous histamine-releasing activity was 6.9 ± 0.3%.

## Results

### MALDI-TOF MS and HPLC analysis of the crude venom extract of *X. appendiculata*

**Fig. 2:**
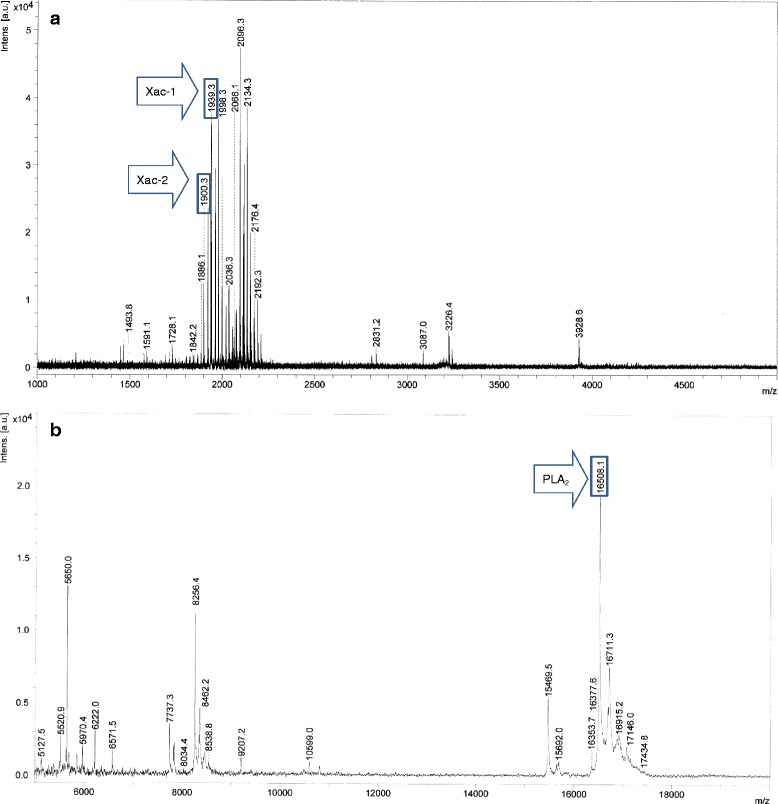
MALDI-TOF MS spectra of the crude venom of X. appendiculata. Positive mode, Matrix: α-CHCA. **a** *m/z* range from 1000 to 5000. *m*/*z* ions: 1939.3, [M + H]^+^ for Xac-1, 1900.3 [M + H]^+^ for Xac-2. **b** *m/z* range from 5000 to 20000. *m*/*z* ions: 16508, [M + H]^+^ ion for PLA_2_

**Fig. 3:**
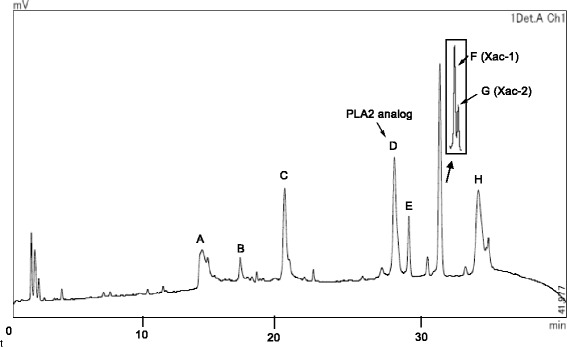
HPLC analysis of the crude venom. Column: COSMOSIL 5C18-AR-300, 4.6 mm × 150 mm (Nacalai Tesque). Eluent: (I) CH_3_CN containing 0.1% TFA and (II) H_2_O containing 0.1% TFA. Elution: linear gradient from (I):(II) = 0.1:99.9 to 60:40. Flow rate, 1.0 mL/min for 45 min. Detection: UV 210 nm. Purity of each fraction was monitored by MALDI-TOF MS. *A*: *m/z* 2066; *B* and *C*: mixture (*m/z* 2066 was mainly detected); *D*: *m*/*z* 16508 (PLA_2_ homolog); E: mixture; F: *m*/*z* 1939.3 (Xac-1); G: *m*/*z* 1900.3 (Xac-2); H: mixture

The crude venom extract of *X. appendiculata* was subjected to MALDI-TOF MS analysis (Fig. 2a and b). The MSPP analysis in a range of *m/z* 1000 ~ 5000 (Fig. 2a) indicated that peptides in a range of *m/z* 1850 ~ 2200 are the major in the venom of *X. appendiculata.* Characteristic ion signal at *m/z* 16508 was observed in a range of *m/z* 5000 ~ 20000 (Fig. 2b). Having these profiles, the crude venom was subjected to HPLC purification with a C18-reversed phase column to provide eight major fractions (A to H) (Fig. 3). Fractions A, D, F, and G included peptides with *m/z* 2066, 16508, 1939.3 and 1900.3, respectively. These molecular ions were originated from the venom because the same *m*/*z* were found in the crude venom analysis. MS analysis of fractions B, C, E, and F showed that these are composed of a mixture of several peptides.

### Peptide sequence of peptides in fractions A, D, F, and G

Edman degradation of fraction F ([M + H]^+^ *m/z* 1939.3) provided a partial peptide sequence – GFVALLKKLPLILKHL – except for the C-terminal amino acid residue. MS/MS analysis (-L/I-L/I-L/I-K-H-L/I-H) indicated that the amino acid residue at the C-terminal was histidine (Additional file 1). Although the sequence was putatively assigned as GFVALLKKLPLILKHLH, the theoretical monoisotopic mass number of GFVALLKKLPLILKHLH-OH (1939.25) differed from the observed mass number (1938.2). These results suggest a possibility of the C-terminal amidation. To prove this possibility, GFVALLKKLPLILKHLH-NH_2_ was prepared and subjected to HPLC analysis to compare retention time. Retention times of the synthetic and naturally occurring peptide were identical. As a result, the peptide of fraction F was determined to be GFVALLKKLPLILKHLH-NH_2_. In a similar manner, the peptide of fraction G was identified as GFVALLKKLPLILKHLP-NH_2_ (Additional file 2).

These peptide sequences were not registered in the NCBI database (http://www.ncbi.nlm.nih.gov/) and Hymenoptera Genome Database (http://hymenopteragenome.org/). Thus, we named these novel peptides Xac-1 (GFVALLKKLPLILKHLH-NH_2_, [M + H]^+^ *m/z* 1939.3) and Xac-2 (GFVALLKKLPLILKHLP-NH_2_, [M + H]^+^ *m/z* 1900.3). Edman degradation analysis of fraction A ([M + H]^+^ *m/z* 2066) was not successfully done, though the reason was unclear. It is speculated that it might be a cyclic peptide with an S-S bond that prevent the Edman analysis. Further sequence analysis is ongoing.

**Fig. 4:**
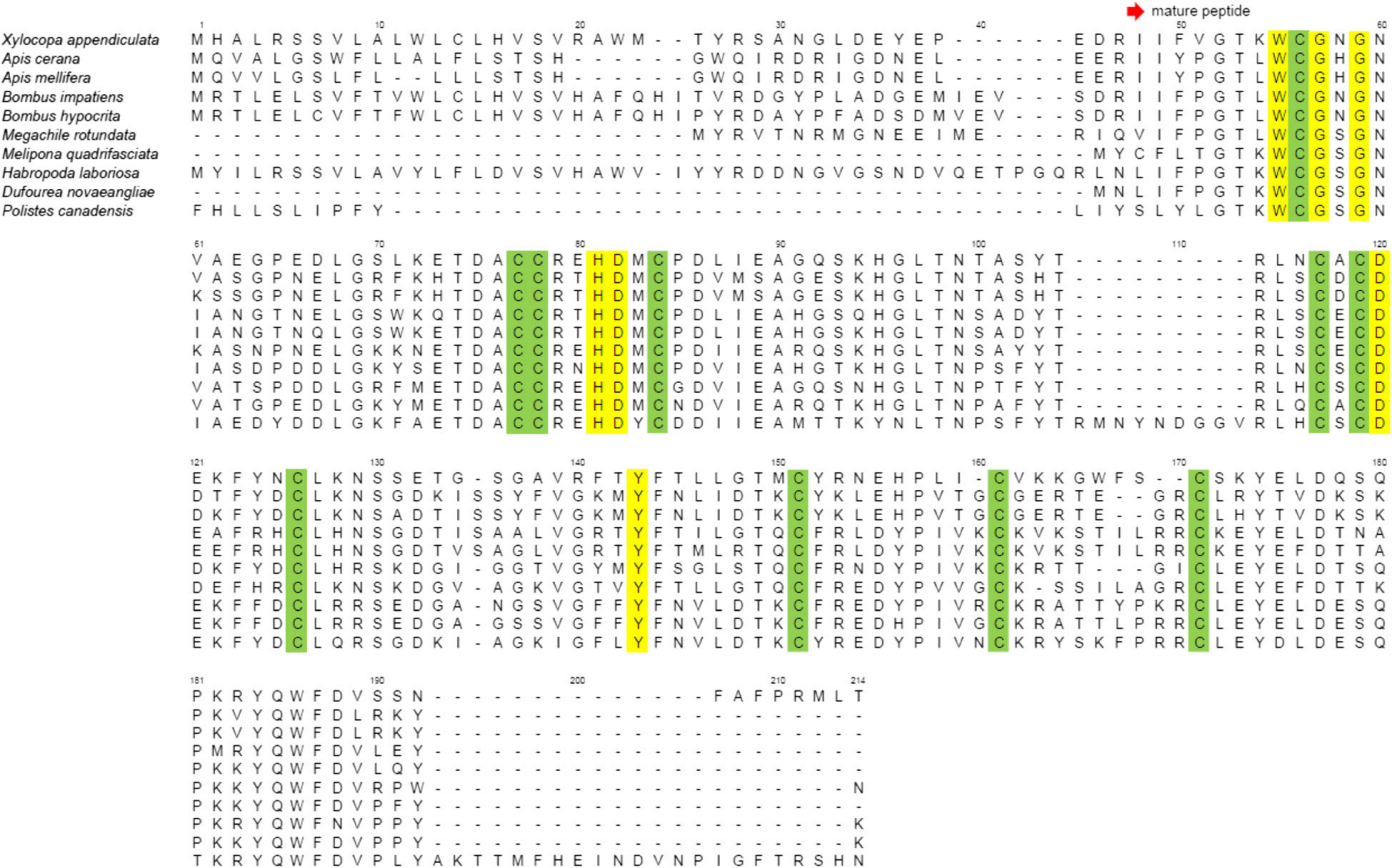
PLA_2_ of *X. appendiculata* and other bees. This alignment file was used to construct the phylogenetic tree shown in Figure 9. Hyphen: alignment gap; yellow: the key amino acid residues for PLA_2_ catalytic activity; green: cysteine. GenBank accession numbers of PLA_2_ are as follows; *Apis cerana* (XP_016913788), *Apis mellifera* (NP_001011614), *B. impatiens* (XP_012248547)*, Bombus hypocrita* (AGW23551), *Megachile rotundata* (XP_012142828), *Melipona quadrifasciata* (KOX69497), *Habropoda laboriosa* (KOC66459), *Dufourea novaeangliae* (KZC10443), and *Polistes canadensis* (XP_014611896)

Edman degradation of the peptide of fraction D provided a partial sequence: IIFVG TKWCG NGNVA EGPED LGSLK E-. Sequence similarity searches showed that the partial sequence conserved a 70% identity with those of PLA_2_s isolated from the bumblebee *Bombus hypocrite* (Apidae: Apinae: Bombini) and the social honeybee *A. mellifera* [18]. We hypothesized that this peptide would be a PLA_2_ homolog and attempted molecular cloning and RACE to elucidate the full nucleotide sequence encoding this peptide (Fig. 4). The resulting sequence (DDBJ/GenBank/EMBL accession no. AB731659) was compared with those of PLA_2_s isolated from bee venom, indicating that the PLA_2_ homolog conserves characteristic amino acid residues associated with the catalytic activity of PLA_2_s of honey and bumblebees [18, 19]. It is speculated that the PLA_2_ of *X. appendiculata* is a product of a post-translational modification due to the fact that the molecular mass number of fraction D ([M + H]^+^ *m/z* 16508) was not identical to that of the peptide estimated by the molecular cloning.

### Physicochemical properties of Xac-1 and Xac-2: helical wheel projection analysis, CD spectroscopy analysis, and liposome leakage assay

**Fig. 5:**
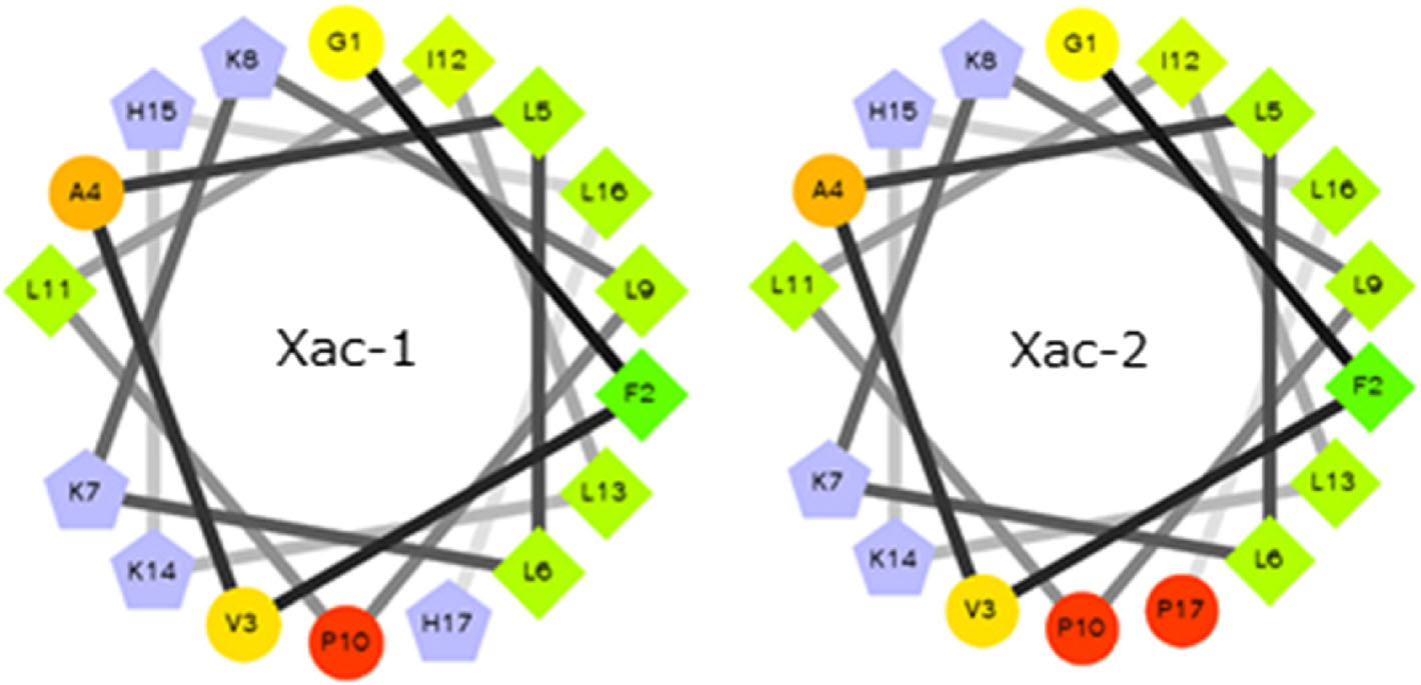
Helical wheel projection of Xac-1 and Xac-2. *Blue*: basic amino acids, others: neutral amino acids

**Fig. 6:**
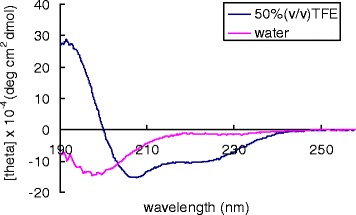
Circular dichroism spectra of 0.2 mM Xac-1 in water and 50%(v/v) aqueous TFE

**Fig. 7:**
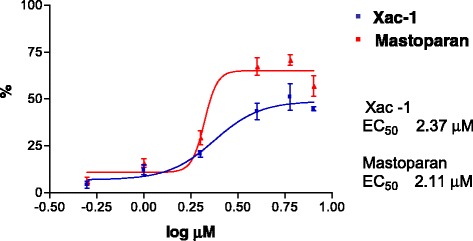
Liposome leakage assay of Xac-1 (*blue*) and mastoparan (*red*)

The helical wheel projection of Xac-1 and Xac-2 was made by the database program (http://www.tcdb.org/progs/?tool=pepwheel) [20]. The results suggest that these peptides possess amphiphilic helical structures in which positively charged amino acid resides, histidines and lysines are arranged on one side and hydrophilic residues on the other side (Fig. 5). To obtain an analytical proof, CD spectra of Xac-1 was measured. Xac-1 exhibited a mostly disordered conformation in aqueous solution whereas a higher α-helical content in 50% TFE solution (Fig. 6). The presence of two negative dichroic bands at 208 and 222 nm was consistent with the preferential formation of α-helix. Subsequently, we analyzed liposome leakage properties of Xac-1 (Fig. 7). Xac-1 revealed liposome degradation activity in which its potency was almost the same as that of mastoparan.

### Biological activities of Xac-1 and Xac-2

Antimicrobial and hemolytic activities of Xac-1 and Xac-2 were examined. Mastoparan (14 amino acid amphiphilic peptides from wasp venom) was selected as a reference peptide because it is a representative amphiphilic peptide that shows antimicrobial and hemolytic activities due to its potent pore forming effects and mast cell degradation activities [7]. In addition, melittin isolated from the venom of *A. mellifera* was used as a reference to compare hemolytic activity [3]. These results are summarized in Table 1. Xac-1 exhibited growth inhibitory effects against *E. coli*, *S. aureus, M. luteus*, and *S. cerevisiae* with MIC values in a range from 1.57 to 6.25 μM. The potency is similar to that of mastoparan. Xac-2 showed almost the same or slightly less potency as Xac-1 on the antimicrobial activities using *M. luteus* and *S. cerevisiae.* Xac-1 and Xac-2 exerted hemolytic activities (37.5 and 23.5% at 100 μM), respectively. These data were compared with those of mastoparan (40.6% at 100 μM) and melittin (91.8% at 10 μM). These results indicated that the potencies of Xac-1 and Xac-2 were close to that of mastoparan, whereas these potencies were much weaker than that of melittin. Bioactive peptides isolated from ant, bee, and wasp have been shown to activate the release of histamines from rat peritoneal mast cells [17]. Both Xac-1 and Xac-2 caused a significant and dose-dependent histamine release. At a concentration of 10 μM, Xac-1 and Xac-2 displayed 58.0 and 53.0% of histamine-releasing activities, respectively. These activities comparable to mastoparan (57.6%), but less effective than melittin (84.8%).

**Table 1:** Biological activities of Xac-1, Xac-2, mastoparan, and melittin

	Antimicrobial activity (MIC)				Hemolytic activity at 100 μM	Histamine releasing activity at 10 μM
	*E. coli* (NBRC 14237)	*S. aureus* (NBRC 12732)	*M. luteus* (NBRC 12708)	*S. cerevisiae* (NBRC 10217)		
Xac 1	3.12 μM	1.57 μM	3.12 μM	6.25 μM	37.5 ± 1.9%	58.0 ± 1.7%
Xac 2	3.12 μM	3.12 μM	6.25 μM	25.0 μM	23.5 ± 1.3%	53.0 ± 4.3%
Mastoparan	6.25 μM	1.57 μM	3.12 μM	6.25 μM	40.6 ± 2.7%	57.6 ± 1.0%
Melittin	–	–	–	–	91.8 ± 1.8%^a^	84.8 ± 10.1%

^a^Hemolytic activities at 10 μM of melittin was indicated

## Discussion

Bees are classified into seven families including more than 16,000 described species [21]. Female bees use their venom for defense when they are exposed to dangers and predators. Bee stings are known to be painful. In contrast to the unpleasant effects of bee toxins in humans, its venom has been utilized as a remedy for centuries and recently has attracted much attention as a promising alternative and preventive medicine for the treatment of arthritis, rheumatism, pain, and cancer, etc. [8, 22]. Although many biologically active peptides and enzymes have been isolated from the venom of social honeybees such as *A. mellifera* and a eusocial bumblebee (*Megabombus pennsylvanicus*), the structure elucidation of the venom substances of carpenter bees has not been well examined except for the Nakajima’s study [23] on the analysis of biogenic amines in the venom of *X. appendiculate.* It revealed that histamine, putrescine and spermidine were detected as major biogenic amines in the venom. Piek [15] predicted that the presence of melittin-like peptides in the venom of *X. violacea* by comparison with biological activities of the crude venom of *X. violacea*, *A. mellifera,* and *Bombus terrestris*. To the best of our knowledge, isolation of the peptide substances in the carpenter venom has not been elucidated yet.

In this study, we found two novel amphiphilic peptides, Xac-1 and Xac-2, and a new PLA_2_ homolog in the venom of *X. appendiculata* for the first time. Our results corroborate that by Nakajima et al. [23] by clearly showing that the venom of *X. appendiculata* is a cocktail of biogenic amines, amphiphilic peptides, PLA_2_ and the molecular constitution resembles those of honeybees and bumblebees. It is supposed that Xac-1 and Xac-2 would be a principle of the melittin-like peptide proposed by Piek [15] since biological activities of Xac-1 and Xac-2 resemble those of melittin.

**Fig. 8:**
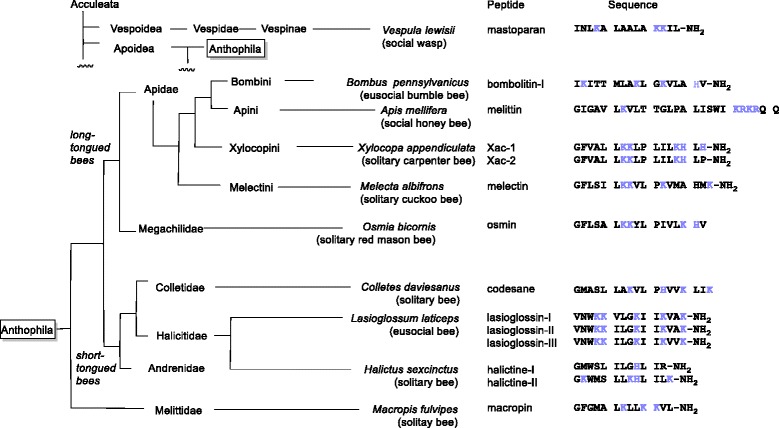
Amphiphilic venom peptides isolated from bees and wasps. The cladogram of bee families is based on Hedtke et al. [21]. *Blue*: basic amino acid residues

Recently, the research interests for venoms has reached other families of solitary and eusocial bees (Fig. 8). These studies have unveiled the distribution of amphiphilic and biologically active peptides such as melectin from *Mellecta albifrons* (Apoidea: Melectini) [24], codesane from *Colletes daviesanus* (Colletidae) [25], osmin from *Osma rufa* (Megachilidae) [26], lasioglossins from *Lasioglossum laticeps* (Halicitidae) [27], halictines from *Halictus sexcinctus* (Halicitidae) [28], macropin from *Macropis fulvipes* (Melittidae) [29] in the bee venom. It is interesting to note that the amino acid sequences of Xac-1 and Xac-2 are similar to those of melectin and osmin isolated from the long-tonged bees, but not to those of bombolitins and melittin isolated from the social bee venom, though carpenter bees, bumblebees and honeybees are closely related. These comparable analyses indicate a possibility that Xac-1, Xac-2, melectin and osmin would be derived from a prototype amphiphilic peptide of the ancestor of solitary bees. On the other hand, melittin, bombolitins, mastoparan may have separately developed during the course of the social evolution. To prove this hypothesis, further research on isolation and biological studies on the bee venom peptides are required.

**Fig. 9:**
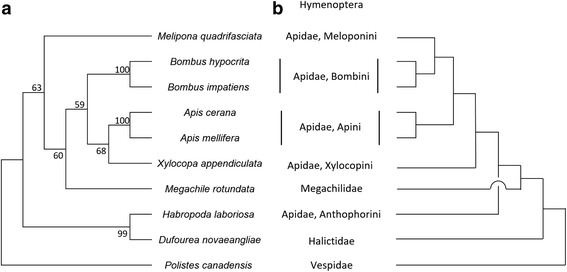
Molecular Phylogenetic analysis by Maximum Likelihood method based on PLA2 amino acid sequences (**a**) and phylogenetic tree proposed by Hedtke et al. [21] (**b**). The evolutionary history was inferred by using the Maximum Likelihood method based on the Whelan And Goldman model [32]. The tree with the highest log likelihood (-1575.2225) is shown. The percentage of trees in which the associated taxa clustered together is shown next to the branches (bootstrap value). Initial tree(s) for the heuristic search were obtained automatically by applying Neighbor-Join and BioNJ algorithms to a matrix of pairwise distances estimated using a JTT model, and then selecting the topology with superior log likelihood value. The rate variation model allowed for some sites to be evolutionarily invariable ([+I], 28.9827% sites). All positions containing gaps and missing data were eliminated. There were a total of 130 positions in the final dataset. Evolutionary analyses were conducted in MEGA7 [33]

PLA_2_ is known to be the main enzyme component of bee venom. Previously, the presence of PLA_2_ in the venom of *Anthophora pauperata* (Apidae) was proposed by biological and hematological studies [30]. To the best of our knowledge, structure analysis of the PLA_2_ of carpenter bees has not been examined yet. We isolated the PLA_2_ of *X. appendiculata* and found that it has a high sequence identity with PLA_2_ of related species such as bumblebees and honeybees (Fig. 4) [31]. We also analyzed the molecular evolution of bee PLA_2_s using database sets (Fig. 9) [32, 33]. Interestingly, PLA_2_ evolution tree did not match with the bee phylogeny that is well-established by large dataset though the characteristic amino acid residues of the PLA_2_ family of honeybees and bumblebees are highly conserved in the PLA_2_ of *X. appendiculata.* Our analysis would contribute to discuss the evolution patterns of PLA_2_s of the bee venoms.

## Conclusion

We have analyzed the venom components of the solitary bee *X. appendiculata* and isolated novel amphiphilic peptides, Xac-1 and Xac-2, and a PLA_2_ homolog. The accurate analysis and structure determination of the venom reveals that it is a cocktail of various biologically active molecules. Our study helps to understand the biological function and molecular diversity of the solitary bee venom components. Additionally, it may aid in the design of biologically active peptides based on the structures of Xac-1 and Xac-2 to develop more potent peptide analogs toward biotechnological and medical applications.

## Additional files

Additional file 1: MS/MS analysis of Xac-1. (DOCX 175 kb)

Additional file 2: MS/MS analysis of Xac-2. (DOCX 157 kb)

## Abbreviations

CD: Circular dichroism
CHCA: α-Cyano-4-hydroxycinnamic acid
HPLC: High performance liquid chromatography
MALDI-TOF MS: Matrix-assisted laser desorption/ionization-time of flight mass spectroscopy
MS/MS: Tandem mass spectrometry
PLA_2_: Phospholipase A_2_
TFA: Trifluoroacetic acid
