# Supplementary figures and images for: Isolation of biologically active peptides from the venom of Japanese carpenter bee, Xylocopa appendiculata
Source: J Venom Anim Toxins Incl Trop Dis. 2017 May 23;23:29. doi: 10.1186/s40409-017-0119-6 (PMC5442655; doi:10.1186/s40409-017-0119-6)

**
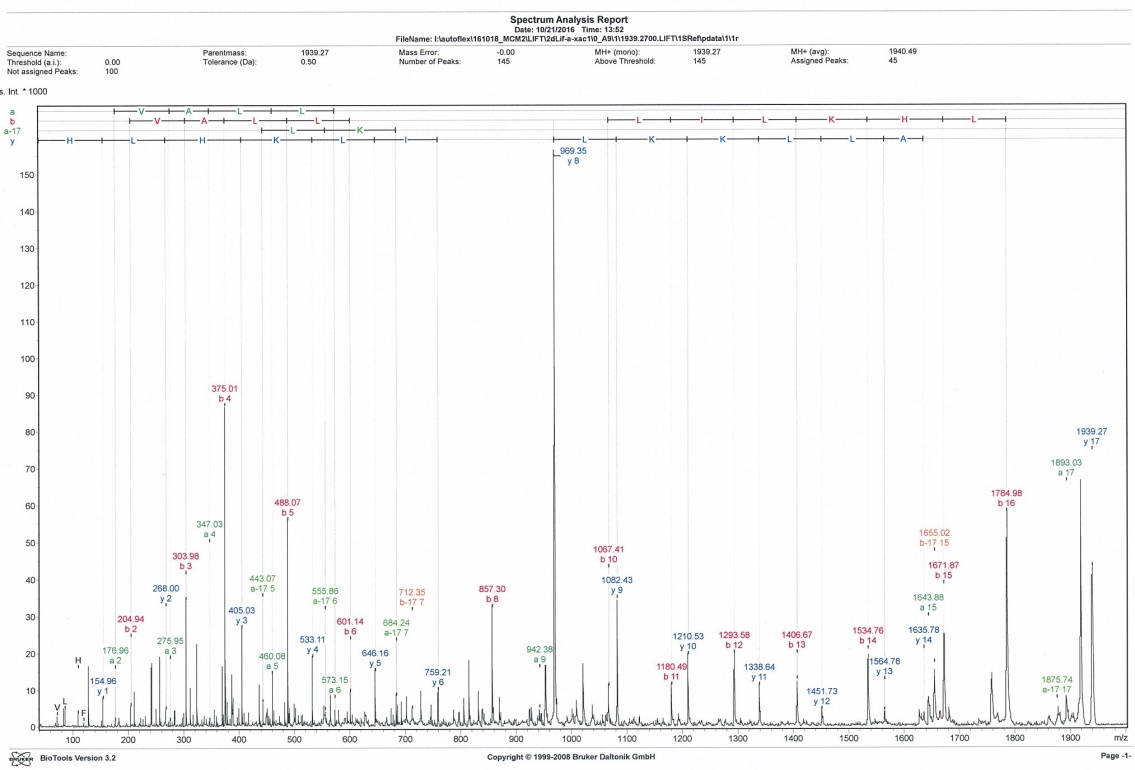
**

**Additional file 1.** MS/MS analysis of Xac-1.

Supplement: Supplementary file 1 — MS/MS analysis of Xac-1. (DOCX 175 kb) [file 40409_2017_119_MOESM1_ESM.docx]

**
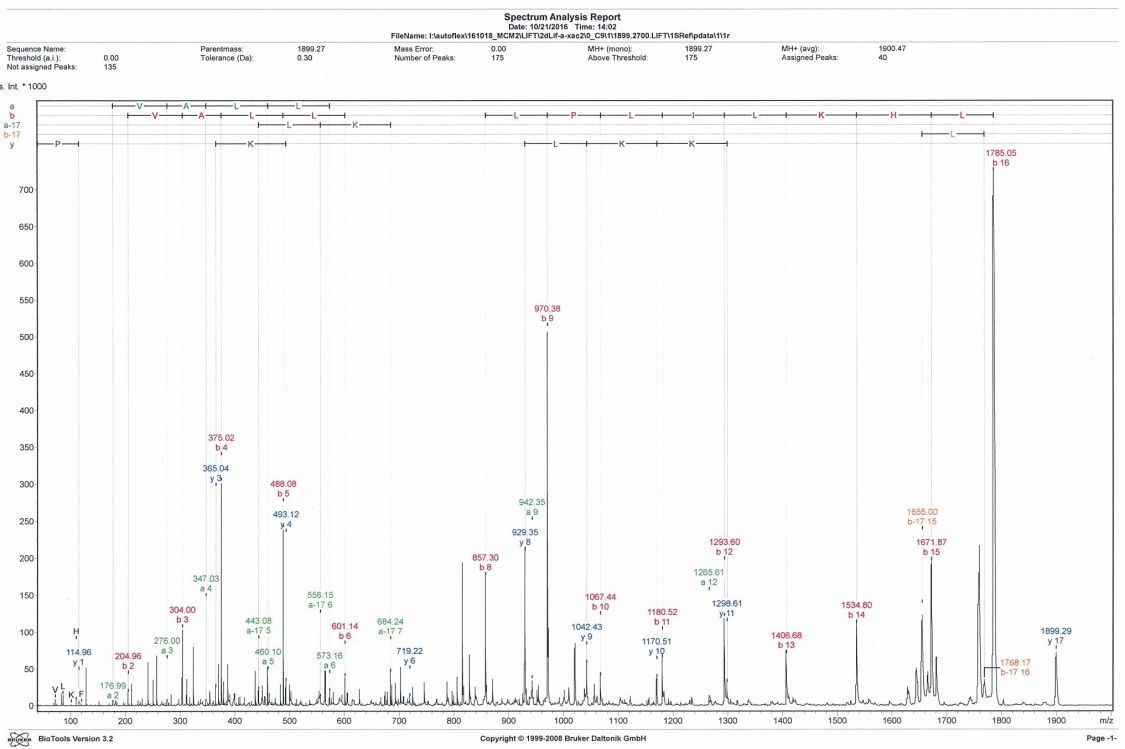
**

**Additional file 2.** MS/MS analysis of Xac-2.

Supplement: Supplementary file 2 — MS/MS analysis of Xac-2. (DOCX 157 kb) [file 40409_2017_119_MOESM2_ESM.docx]
